# Expression analysis of zinc-metabolizing enzymes in the saliva as a new method of evaluating zinc content in the body: two case reports and a review of the literature

**DOI:** 10.1186/s13256-024-04463-w

**Published:** 2024-04-04

**Authors:** Ken-ichiro Sakata, Ayako Hashimoto, Taiho Kambe, Jun Sato, Noritaka Ohga, Yutaka Yamazaki, Masahide Koyachi, Itagaki Tatsuki, Mai Okada, Okura Taro, Hiroshi Hikasa, Yoshimasa Kitagawa

**Affiliations:** 1 Department of Oral Diagnosis and Medicine, Division of Oral Pathobiological Science, Faculty of Dental Medicine and Graduate School of Dental Medicine, Hokkaido University, Sapporo, Japan; 2 Department of Food and Nutrition, Faculty of Home Economics, Kyoto Women’s University, Kyoto, Japan; 3 Department of Applied Molecular Biology, Division of Integrated Life Science, Graduate School of Biostudies, Kyoto University, Kyoto, Japan; 4 Department of Gerodontology, Division of Oral Health Science, Faculty of Dental Medicine and Graduate School of Dental Medicine, Hokkaido University, Sapporo, Japan; 5 Department of Oral Pathobiological Science and Surgery, Tokyo Dental College, Tokyo, Japan

**Keywords:** Zinc-metabolizing enzymes, Serum zinc, Saliva, Total ALP activity

## Abstract

**Background:**

The activity level of alkaline phosphatase, a zinc-requiring enzyme in the serum, is used to indicate zinc nutritional status; however, it does not correlate with serum zinc levels or subjective symptoms of taste disorder in many cases. Hence, this study focused on the total activity of alkaline phosphatase, a zinc-requiring enzyme. The total alkaline phosphatasa activity level in the saliva was measured before and after zinc supplementation, and the results were compared with serum zinc levels.

**Case presentation:**

This study included patients with hypozincemia, specifically a patient with zinc-deficient taste disorder (patient 1: a 69-year-old Japanese woman) and a patient with glossodynia with zinc deficiency (patient 2: an 82-year-old Japanese woman). Saliva samples were collected, and blood tests were performed before and after zinc supplementation. Subjective symptoms and serum zinc levels were simultaneously evaluated. Zinc supplementation was performed using zinc acetate hydrate or Polaprezinc.

**Conclusions:**

Total alkaline phosphatase activity levels were found to be associated with serum zinc levels and subjective symptoms. A further study with a higher number of patients is necessary to confirm whether total alkaline phosphatase activity levels more accurately reflect the amounts of zinc in the body than serum zinc levels.

## Background

**Fig. 1:**
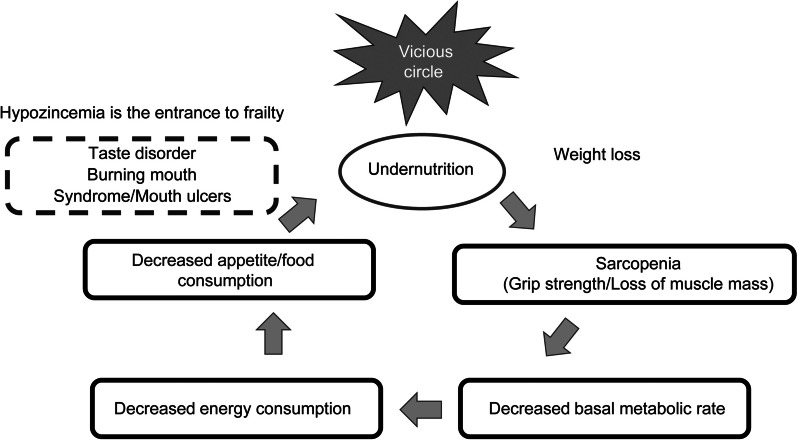
Hypozincemia leads to frailty

Zinc deficiency in humans was recognized for the first time in 1961 [1]. Studies have reported that zinc deficiency is associated with taste disorder and glossodynia [2–4]. There are several other symptoms, such as bedsores, skin symptoms, delayed wound healing, fetal growth restriction, chronic diarrhea, anemia, and altered mental status [5]. The Zincage Project (research on the relationship between health/aging and zinc), an epidemiological survey in Europe, has shown that health status of older adults is positively associated with the amount of zinc in the body [6]. In the basic research field, zinc is considered an essential trace metal element, with approximately 2 g being present in the body. Zinc plays a role as the active center of 3000 types of enzymes and bioactive substances [7]. Recent human genome decoding projects have indicated that approximately 10% of genes have zinc-binding domains. Hence, researchers have focused their attention on the relationship between zinc and diverse physiological functions, including zinc transporters and zinc signals [8]. Sequelae of oral diseases caused by zinc deficiency lead to a frailty cycle. This cycle stems from appetite loss and decreased food consumption, causing undernutrition. This state results in weight loss and sarcopenia onset (Fig. 1).

To address this issue, accurate measurement of the zinc content in the body is required in clinical practice, highlighting the need to develop a method for accurate and rapid diagnosis of zinc deficiency. Zinc deficiency is known as the main factor contributing to taste disorder [9]. However, it is not well known that zinc deficiency can also cause several oral diseases, such as mouth ulcers and glossodynia, posing a diagnostic and treatment challenge in many cases [10]. Serum zinc levels are a commonly used indicator of zinc assessment in clinical practice. However, they do not accurately reflect the zinc content in the body, because they account for only less than 0.1% of the total zinc in the body [3]. Furthermore, they exhibit circadian rhythms and are easily influenced by several conditions. Owing to such disadvantages, they are not considered an absolute indicator [11]. In this study, we attempted to identify an indicator other than serum zinc levels for accurately measuring the amount of zinc in the body, focusing our attention on zinc-requiring enzymes, which show zinc-dependent activity. Zinc-requiring enzymes are activated after binding to zinc. The activity level of alkaline phosphatase (ALP), a zinc-requiring enzyme in the serum, is used as an indicator of zinc nutritional status; however, it does not correlate with serum zinc levels or subjective symptoms of taste disorder in many cases. To accurately evaluate the zinc nutritional status, we shifted our focus from serum ALP to total alkaline phosphatase [12, 13]. Four ALP isozymes are  broadly expressed in various cells (Table 1). Previous studies using cultured cells and animals have shown that ALP, an enzyme that is activated by coordinating zinc at the active center, plays a role in suppressing inflammation by hydrolyzing adenosine triphosphate (ATP), which serves as a danger signal extracellularly, into adenosine. The results demonstrate that ALP is a zinc-requiring enzyme (zinc marker enzyme), enabling the accurate measurement of zinc content in the body [14].

**Table 1:** ALP isoform

Gene	Protein	Isoforms	Localization	Functions
ALPL	ALP, tissue-nonspecific isozymes (TNAP)	Bone-specific TNAP	Skeletal tissue, kidney, and other cell types	Skeletal mineralization Vitamin B_6_ metabolism
		Liver-specific TNAP	Liver	Unknown
ALPI	ALP, intestinal-type (IAP)	Unknown	Mainly duodenum	Involved in fat absorption Detoxification of LPS Regulation of intestinal microbiota
ALPP	ALP, placental-type (PALP)	Unknown	Syncytiophoblasts Several tumors	Tumor marker Detoxification of bacterial toxins
ALPPL2	ALP, placental-like	Unknown	Testis, malignant trophoblasts Testicular cancer	Unknown

ALP: alkaline phosphatase, TNAP: tissue-nonspecific isozymes, IAP: ALP, intestinal-type, PALP: ALP, placental-type, LPS: lipopolysaccharide

Objective: Intervention is required to break the frailty cycle; however, the following questions remain unsolved: “Which indicators should be used for zinc supplementation?”, “How long should zinc supplementation be continued?”, and “How much zinc is required for the supplementation?” We raised the possibility of conducting translational research in which basic scientific findings are translated into clinical practice, with the aim of attaining a society where “older adults can enjoy meals.” This study aimed to evaluate total ALP activity levels in the saliva, serum zinc levels, and subjective symptoms before and after zinc supplementation in patients with oral diseases caused by zinc deficiency.

## Case presentation

### Patients and methods

Saliva samples were used because they can be collected repeatedly in a noninvasive manner. They were collected before and after zinc supplementation, and subjective symptoms of taste disorder and serum zinc levels were simultaneously evaluated. Saliva sample collection and blood tests were performed only in the morning. The gustatory testing (whole mouth) results are as follows: Four types of taste solutions [sucrose (S), sodium chloride (NaCl; N), tartaric acid (T), and quinine hydrochloride dihydrate (Q)] were dropped on the dorsum of the tongue at the following concentrations (No. 1: 2.9 μmol, No. 1.5: 5.8 μmol, No. 2: 8.8 μmol, No. 2.5: 17.5 μmol, No. 3: 29.2 μmol, No. 4: 58.4 μmol, and No. 5: 2337 μmol), and cognitive thresholds were tested by tasting the samples within 2–3 seconds. Taste perception threshold testing followed the criteria in the table below, and when No. 5 could not be recognized, it was considered taste loss. The efficacy of zinc supplementation in patients with taste disorder and those with burning mouth syndrome (BMS) was evaluated using the Visual Analog Scale [15] and Numerical Rating Scale [16], respectively. Zinc content was measured using ACCURAS AUTO Zn (Shino-Test Corporation, Osaka, Japan).

Zinc was measured using the Acurus Auto Zn (Shino-Test Corporation, Tokyo, Japan) based on the colorimetric method using an automated analyzer (Hitachi 7700 series; Hitachi High-technology Corporation, Tokyo, Japan). Measurement of ALP activity in saliva was performed as follows: 5 µl of saliva was used to measure ALP activity. A total of 100 μl of substrate solution [2 mg/ml disodium *p*-nitrophenylphosphate hexahydrate (*p*NPP); Wako Pure Chemicals in 1 M diethanolamine buffer pH 9.8 containing 0.5 mM MgCl_2_] was added and incubated at 37 ℃ for 1 hour. The released *p*-nitrophenol product was quantified by measuring the absorbance at 405 nm.

Calf intestinal ALP (Promega) was used to generate a standard curve. This study was approved by the Hokkaido University Hospital Independent Clinical Research Review Committee (Approval No. 019-0337) and approved by the Ethics Committee of Kyoto, University Graduate School and Faculty of Medicine (Approval No. R3275).

All study procedures were performed in accordance with the principles of the Declaration of Helsinki. Written informed consent was obtained from all participants.

### Cases

**Fig. 2.**
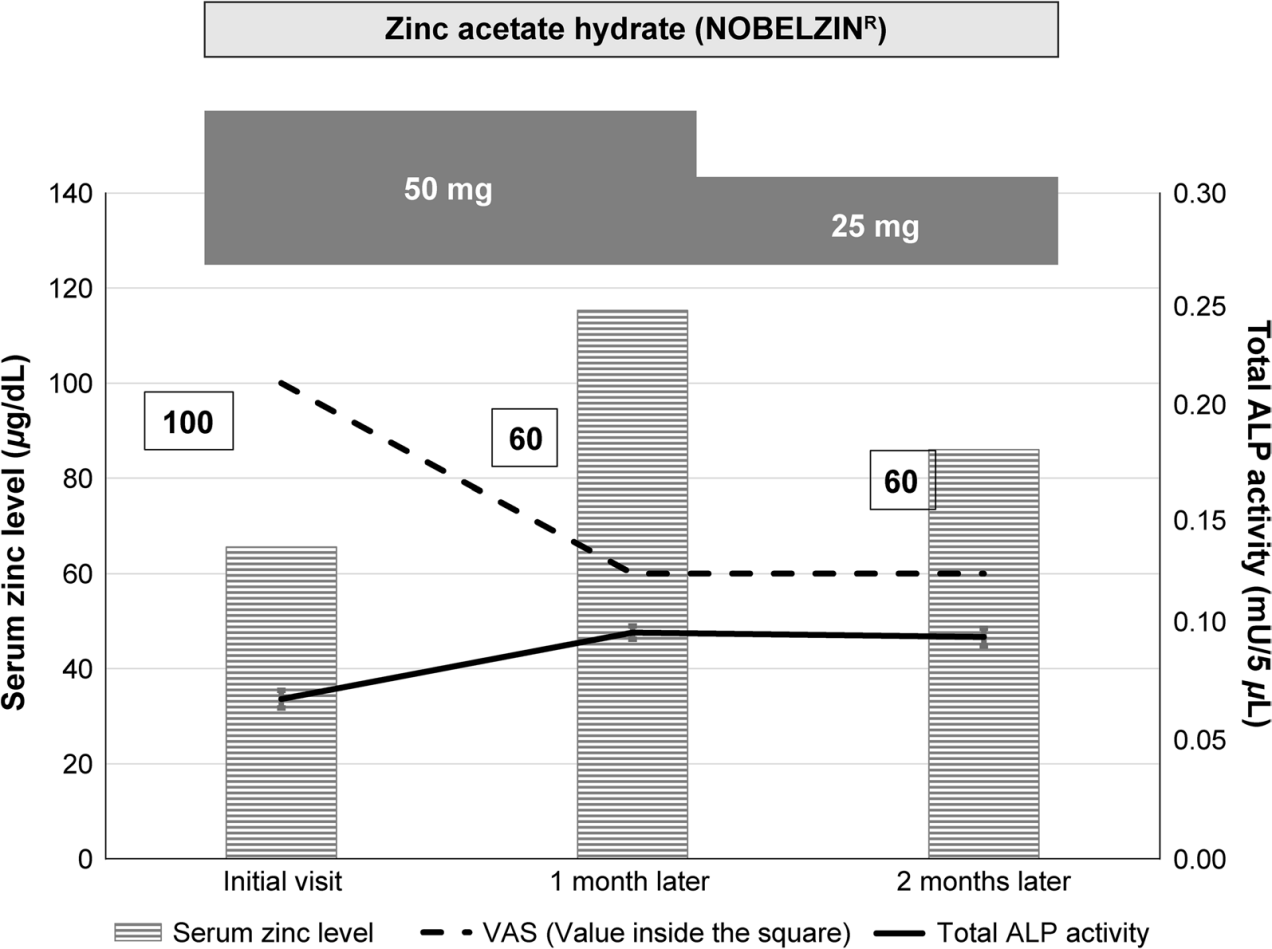
69-year-old woman with taste disorder

Two patients experiencing hypozincemia were included. Patient 1 was a 69-year-old Japanese woman (Fig. 2). The patient presented to the Dentistry Center, Hokkaido University Hospital, with the chief complaint of hypogeusia. In addition to routine intraoral examination, the patient received gustatory testing, oral bacteria testing, blood tests, and chewing gum test. Intraoral examination showed mild xerostomia and tongue coating. The results of gustatory testing (whole mouth) were sweet (2.5), salty (4), sour (2), and bitter (2), indicating hypogeusia. Oral bacteria testing was unremarkable. The volume of saliva collected using the chewing gum test was 17.8 mL. Blood tests at the initial visit showed a serum zinc level of 65.6 μg/dL. Based on this low serum zinc level, the patient was diagnosed with zinc-deficient taste disorder. The total ALP activity level was 0.072 mU/5 μL. Zinc supplementation was continued for 2 months using zinc acetate hydrate. After the patient orally took zinc acetate hydrate 50 mg for 1 month, the serum zinc level improved to 115.3 μg/dL, and the total ALP activity level increased to 0.102 mU/5 μL. After the zinc acetate hydrate dose was reduced to 25 mg, the serum zinc level slightly decreased to 86 μg/dL. However, the total ALP activity level remained almost the same at 0.1 mU/5 μL.

**Fig. 3:**
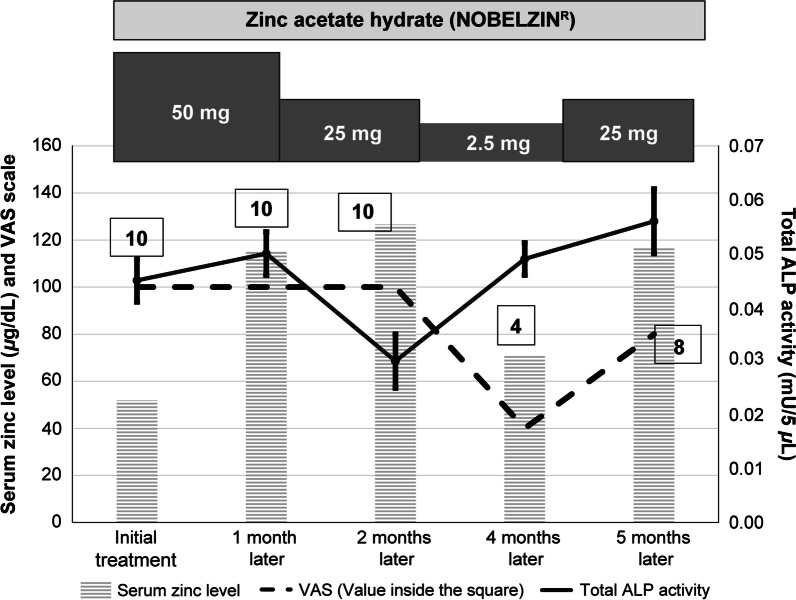
An 82-year-old woman with glossodynia

Patient 2 was an 82-year-old Japanese woman (Fig. 3). The patient presented to the Dentistry Center, Hokkaido University Hospital, with the chief complaint of glossodynia. In addition to routine intraoral examination, she received gustatory testing, oral bacteria testing, and blood tests. Intraoral examination showed mild tongue coating. The results of gustatory testing (whole mouth) were sweet (1.5), salty (2.5), sour (2.5), and bitter (2), indicating hypogeusia. Results of oral bacteria testing were positive for *Candida albicans*. Administration of amphotericin B gargles eradicated *C. albicans*. However, glossodynia persisted after the eradication; therefore, the patient was diagnosed with BMS secondary to zinc deficiency. Blood tests at the initial visit showed a serum zinc level of 51.9 μg/dL. On the basis of this low serum zinc level, the patient was diagnosed with BMS secondary to zinc deficiency. The total ALP activity level was 0.045 mU/5 μL. Zinc supplementation was continued for 5 months using zinc acetate hydrate. After the patient orally took zinc acetate hydrate 50 mg for 1 month, the serum zinc level improved to 113.3 μg/dL, and the total ALP activity level increased to 0.05 mU/5 μL. The patient received a reduced zinc acetate hydrate dose of 25 mg for the subsequent month of zinc supplementation, and the serum zinc level and total ALP activity level 2 months after treatment initiation were 126.6 μg/dL and 0.03 mU/5 μL, respectively. Zinc acetate hydrate 25 mg was administered every other day over the third and fourth months after the initiation of zinc supplementation. Serum zinc and total ALP activity levels 4 months after treatment initiation were 71.3 μg/dL and 0.049 mU/5 μL, respectively. Zinc acetate hydrate 50 mg was administered for the fifth month after the initiation of zinc supplementation. Serum zinc and total ALP activity level 5 months after treatment initiation were 116.6 μg/dL and 0.056 mU/5 μL, respectively. The serum zinc level decreased transiently, but the total ALP activity level remained almost the same.

## Discussion and conclusions

### Unstable serum zinc levels

Currently, zinc supplementation is performed on patients with taste disorder and glossodynia due to zinc deficiency based on serum zinc levels. Our previous study showed that the mean serum zinc level was 72 μg/dL in the taste disorder group and 74 μg/dL in the control group, with no relationship between taste disorder and serum zinc levels [17]. The proportion of patients with zinc deficiency in the taste disorder group was significantly higher (*p* < 0.05) only when a serum zinc level of < 60 μg/dL (that is, severe zinc deficiency) was used to identify patients with zinc deficiency. Possible explanations for the absence of an association between clinical symptoms and serum zinc levels include: (1) Serum zinc levels do not accurately reflect the zinc content in the body; (2) serum zinc levels exhibit a large fluctuation of circadian rhythms and are easily influenced by meals, stress, and hormone conditions, therefore making them problematic as an indicator of zinc deficiency; and (3) differences in serum zinc levels between laboratories (serum zinc levels tend to show a higher value in outsourcing examination than in-hospital examination owing to hemolysis). These disadvantages suggest that serum zinc levels may not reflect the bioavailability of zinc in the body in some cases. Thus, selecting an appropriate sample and developing a method to analyze the sample are important issues.

### Taste disorder and zinc

The relationship between taste disorder and zinc deficiency plays an important role in regenerating taste bud cells. Taste buds, or taste sensors, are actively regenerated approximately every 10 days. Normal regeneration of taste bud cells is required to correctly recognize different tastes. Insufficient regeneration of taste bud cells due to zinc deficiency is associated with a decrease in the number of these cells, causing impairment of taste sensor function. Zinc plays an important role in protein formation that is required for cell regeneration and transmission of genetic information.

### Glossodynia and zinc

Glossodynia is a traditionally well-known symptom of hypozincemia in clinical practice; however, its evident cause is unknown [18]. Zinc exerts analgesic effects when administered locally to the spinal nerves or peripheral nerves or systemically in patients with chronic pain in areas other than in the oral area, renal dysfunction, anemia, and itchy sensation on the skin [19, 20]. The mechanism of this analgesic effect was reported in a study in 2011 [21]. In the said study, increased sensitivity to pain was observed in knockout mice whose zinc ion binding to the NMDA-type glutamate receptor was inactivated. The authors reported that C fibers were particularly affected as compared with Aδ fibers, resulting in difficulty in suppressing chronic pain. Chronic glossodynia signals are transmitted to the brain mainly via C fibers, suggesting that the association between hypozincemia and glossodynia may be mediated by NMDA receptors, although the details remain to be clarified.

### Total ALP activity levels

Both patients experienced a transient decrease in serum zinc levels, but total ALP activity levels did not change. Total ALP activity is involved in the dephosphorylation of various physiological substrates and has vital physiological functions, including extraskeletal functions such as neuronal development, detoxification of lipopolysaccharide, an anti-inflammatory role, bile pH regulation, and maintenance of the blood–brain barrier. Total ALP activity has also been reported to be involved in cardiovascular calcification, chronic kidney disease, and type 2 diabetes [22]. In particular, it is a logical clinical target to attenuate vascular calcification. Total ALP activity has gained attention as a clinical marker in several diseases. The relationship between total ALP activity levels and changes in zinc content in the body has been evaluated only in studies using cultured cells and animals. Thus, this is the first study to report the relationship in humans. Furthermore, this study used saliva samples; therefore, the results are extremely useful because these samples can be collected repeatedly in a noninvasive manner. Further cross-sectional or cohort studies are required to clarify the relationship between serum zinc levels and zinc-requiring enzymes, including total ALP activity, to interrupt the disease cycle due to zinc deficiency and improve the health status. This study suggests that total ALP activity may be a new diagnostic marker alternative to serum zinc levels. Furthermore, it may be used as an indicator when assessing the efficacy of zinc supplementation.

## Data Availability

No new data were created or analyzed in this study. Data sharing is not applicable to this article.

## Abbreviation

ALP: Alkaline phosphatase
